# Rethinking Probability of Success as Bayes Utility

**DOI:** 10.1002/bimj.70067

**Published:** 2025-07-14

**Authors:** Fulvio De Santis, Stefania Gubbiotti, Francesco Mariani

**Affiliations:** ^1^ Department of Statistical Sciences Sapienza University of Rome Rome Italy

**Keywords:** assurance, Bayesian power, Bayes risk, clinical trials, sample size determination

## Abstract

In the hybrid frequentist‐Bayesian approach, the probability of success (PoS) of a trial is the expected value of the traditional power function of a test with respect to a design prior assigned to the parameter under scrutiny. However, this definition is not univocal and some of the proposals do not lack of potential drawbacks. These problems are related to the fact that such definitions are all based on the probability of rejecting the null hypothesis rather than on the probability of choosing the correct hypothesis, be it the null or the alternative. In this article, we propose a unifying, decision‐theoretic approach that yields a new definition of PoS as the expected utility of the trial (u‐PoS), that is, as the expected probability of making the correct choice between the two hypotheses. This proposal shows a conceptual advantage over previous definitions of PoS; moreover, it produces smaller optimal sample sizes whenever the design prior assigns positive probability to the null hypothesis.

## 1 Introduction

The design of clinical trials is commonly based on the concept of *probability of success* (PoS). However, the definition of PoS is not univocal. Originally, PoS has been defined as the power of a frequentist test, that is, the probability of rejecting the null hypothesis, evaluated at a specific value of the parameter of interest under the alternative hypothesis, typically the guessed true underlying treatment effect (or effects difference). In practice, there might be uncertainty about such design value and, in these cases, the traditional conditional power could be unable to provide an adequate indication of the probability that the trial will end up successfully (Spiegelhalter et al. 2004; Spiegelhalter and Freedman 1986). Several alternative methods have been proposed to bypass such a difficulty. O'Hagan and Stevens (2001) and O'Hagan et al. (2005) introduce the idea of computing PoS as the unconditional probability of rejecting the null hypothesis, that is, as the expected value of the power function with respect to a *design prior* distribution on the parameter space. This “prior distribution expresses *uncertainty* about the fixed but unknown true value […] corresponding to the specified treatment effect […] and describes the relative plausibility of different parameter values” (O'Hagan et al. 2005). The resulting hybrid frequentist‐Bayesian quantity is commonly called *assurance*. But even the definition of assurance is not untroubled and this fact has led to additional alternative versions of PoS. Liu (2010), for instance, notes that assurance is the sum of two probabilities: the probability of correctly rejecting the null hypothesis and that of incorrectly rejecting it. This means that it contains a term that depends on the type I error of the test (i.e., the probability of a wrong decision). For this reason, the author proposes alternative definitions of PoS, that he names *extended Bayesian expected powers*. Kunzmann et al. (2021) review and discuss the many alternative ways to summarize the power function that have been gathering up in the literature over the years. Their analysis points out the strong diffraction in the definition of PoS.

A problem of some of the existing proposals is that, as n goes to infinity, their limiting values depend on the selected prior distribution and they might be smaller than one. For instance, if the design prior assigns a nonnegligible probability to the null, the sequence of assurance probabilities tends to the probability of the alternative hypothesis under the design prior, that is strictly less than one. This fact should be carefully taken into account when a specific definition of PoS is used for sample size determination (Brutti et al. 2008; De Santis and Gubbiotti 2022; Eaton et al. 2013).

Starting from the literature just briefly summarized, the goal of the present article is threefold. We look for a measure of success of a trial, that is: (1) endowed with a formal decision‐theoretic justification; (2) uncontroversial, that is, independent of the probability of incorrectly rejecting the true null hypothesis; and (3) asymptotically equal to one as the sample size goes to infinity, regardless of the design scenario. In this regard, we proceed as follows. First, we propose a unifying definition of PoS based on a decision‐theoretic formulation of the problem. As measure of success of an experiment, this approach naturally yields the average probability of selecting the correct hypothesis, whether it is the alternative or the null. This quantity, that we call u‐PoS (where u‐ reminds the approach based on Bayes *utility*), is particularly appropriate for those trials where the chances that the null hypothesis is true are not a priori excluded. Conversely, when the design prior is fully concentrated on the parameter values of the alternative hypothesis, u‐PoS coincides with assurance. Second, unlike assurance, u‐PoS avoids the problem of mixing‐up expectations of type I error and probability of correct rejection of the null hypothesis. Third, the proposed measure tends to one as n increases for any choice of the design prior distribution.

The article is structured as follows. Section 2 illustrates the decision‐theoretic framework that leads to u‐PoS. In Section 2.1, we sketch the formal relationships between u‐PoS and the main previous definitions. Section 2.2 shows how all the main quantities here discussed can be numerically approximated by a simple Monte Carlo computation that exploits draws from a common design prior. Sample size determination criteria based on u‐PoS and its competitors are defined in Section 2.3. In Section 3, the methodology is applied to normal models and all the ideas are illustrated in an extended example based on a real trial (Bittl and He 2017; Farkouh et al. 2012) where coronary artery bypass graft (CABG) is compared with percutaneous coronary intervention (PCI) in diabetic patients with multivessel coronary artery disease. We consider several alternative design scenarios, that are formalized using both unimodal (Section 3.1) and mixture priors (Section 3.2). Specifically, the latter approach, which yields bimodal design priors, has been proposed in the literature to reflect the uncertainty that a new treatment will be clinically effective, as it is often felt appropriate for the early stage of development (Temple and Robertson 2021). Simulation results and plots can be easily reproduced referring to the Supporting Information and using the Shiny App uPoS available at https://6kp5ow‐francesco‐mariani.shinyapps.io/uPoS/. Numerical comparison of optimal sample sizes obtained using alternative methods shows that u‐PoS yields smaller sample sizes when the design prior assigns nonnegligible probability to the null hypothesis. Finally, Section 4 reports some closing remarks.

## 2 Methodology

Let θ denote an unknown true treatment effect or effects difference and consider the testing problem $H_{0}:\theta \in \Omega_{0}$ vs. $H_{1}:\theta \in \Omega_{1}$, where $\left\{\Omega_{0},\Omega_{1}\right\}$ is a partition of the parameter space Ω⊆R. Let $X_{n}=\left(X_{1},X_{2},\text{…},X_{n}\right)$ denote the random values of a clinical trial endpoint X and let f(·|θ) be the probability mass or density function of $X_{n}$. Let $x_{n}$ be the corresponding observed values, that is, the data that can be used for inference on θ. Under a decision‐theoretic framework, a test statistic is a decision function associated to a partition $\left\{X_{0},X_{1}\right\}$ of the sample space X, defined as follows:

$$\delta_{n}=\delta \left(x_{n}\right)=\left\{\begin{array}{cc} a_{0} & \;x_{n}\in X_{0},\\ a_{1} & \;x_{n}\in X_{1}, \end{array}\right.$$

where $a_{i}$ means accepting $H_{i}$, i=0,1 and where $X_{0}$ and $X_{1}$ are the acceptance and the rejection regions of $H_{0}$, respectively. Assume that the consequence of selecting $H_{i}$, that is, that $\delta \left(x_{n}\right)=a_{i}$, is quantified by the 0−1 *utility function*

(1) $$U\left(\theta ,a_{i}\right)=\left\{\begin{array}{cc} 1 & \;\theta \in \Omega_{i}\\ 0 & \;\theta \in \Omega_{i}^{c} \end{array}\right.=I_{\Omega_{i}}\left(\theta \right),\;i=0,1,$$

where $\Omega_{i}^{c}=\Omega \setminus \Omega_{i}$. This utility function implies a symmetric attitude toward the consequences of accepting/rejecting the hypotheses. Other and more general utility functions could be considered (Parmigiani and Inoue 2009) to formalize unbalanced consequences of the two actions. However, we focus on (1) which yields sensible results whose interpretation is straightforward.

In the standard frequentist setup, the quality of the random decision $\delta (X_{n})$ is evaluated by the *expected utility function*, that is,

$$U\left(\theta ,\delta_{n}\right)=E_{f}\left[U(\theta ,\delta \left(X_{n}\right))\right]=U_{n}\left(\theta \right),$$

where $E_{f}$ denotes the expected value with respect to the sampling distribution f(·|θ). The expected utility function $U_{n}\left(\theta \right)$ provides the pre‐experimental average utility of $\delta_{n}$ as θ varies in Ω. It is straightforward to check that

(2) $$\begin{array}{ccc} U_{n}\left(\theta \right) & = & \left\{\begin{array}{cc} 1-\alpha_{n}\left(\theta \right), & \;\theta \in \Omega_{0}\\ 1-\beta_{n}\left(\theta \right), & \;\theta \in \Omega_{1} \end{array}\right.\\ & = & \;\left[1-\alpha_{n}\left(\theta \right)\right]\;I_{\Omega_{0}}\left(\theta \right)+\left[1-\beta_{n}\left(\theta \right)\right]\;I_{\Omega_{1}}\left(\theta \right), \end{array}$$

where

$$\alpha_{n}\left(\theta \right)=P_{f}\left(X_{n}\in X_{1}\;|\;H_{0}\right)\;\;\text{and}\;\;\beta_{n}\left(\theta \right)=P_{f}\left(X_{n}\in X_{0}\;|\;H_{1}\right)$$

are the type I and type II probability error functions computed with respect to f(·|θ). Recalling that the power function of the test is

(3) $$\begin{array}{ccc} \eta_{n}\left(\theta \right) & = & \left\{\begin{array}{cc} \alpha_{n}\left(\theta \right), & \;\theta \in \Omega_{0}\\ 1-\beta_{n}\left(\theta \right), & \;\theta \in \Omega_{1} \end{array}\right.\\ & = & \alpha_{n}\left(\theta \right)\;I_{\Omega_{0}}\left(\theta \right)+\left[1-\beta_{n}\left(\theta \right)\right]\;I_{\Omega_{1}}\left(\theta \right), \end{array}$$

then

(4) $$\begin{array}{ccc} U_{n}\left(\theta \right) & = & \left\{\begin{array}{cc} 1-\eta_{n}\left(\theta \right), & \;\theta \in \Omega_{0}\\ \eta_{n}\left(\theta \right), & \;\theta \in \Omega_{1} \end{array}\right.\\ & = & \left[1-\eta_{n}\left(\theta \right)\right]I_{\Omega_{0}}\left(\theta \right)+\eta_{n}\left(\theta \right)I_{\Omega_{1}}\left(\theta \right). \end{array}$$

From (2), we can notice that the sum that defines $U_{n}$ involves two expected utilities: $\left[1-\alpha_{n}\left(\theta \right)\right]$ under $H_{0}$ and $\left[1-\beta_{n}\left(\theta \right)\right]$ under $H_{1}$. Conversely, Equation (3) shows that the power function $\eta_{n}\left(\theta \right)$ mixes up the type I error function $\alpha_{n}\left(\theta \right)$ and the expected utility $\left[1-\beta_{n}\left(\theta \right)\right]$ (Liu 2010). This is a consequence of $\eta_{n}\left(\theta \right)$ being the probability of rejecting $H_{0}$, whereas $U_{n}\left(\theta \right)$ takes into account both the probabilities of correct rejection *and* of correct acceptance of $H_{0}$. In other words: $U_{n}\left(\theta \right)$ is the probability of making the correct decision in a test, whatever it is. Therefore, we suggest to use $U_{n}\left(\theta \right)$, instead of $\eta_{n}\left(\theta \right)$, to define PoS (see Section 2.1).

From a frequentist perspective, the quality of the experiment can be assessed in terms of $U_{n}\left(\theta_{d}\right)$, where $\theta_{d}$ is a design value. As discussed in O'Hagan et al. (2005), $\theta_{d}$ can be either “the minimal effect which has clinical relevance” or “the anticipated effect of the new treatment.” However, this conditional approach ignores uncertainty on θ. Conversely, one can specify a *design prior* π(·) on the parameter, that is now considered as a random variable and denoted by Θ. This prior expresses uncertainty about the unknown true value at the design stage and should not be confused with the so‐called *analysis prior* that is the one used to combine pre‐experimental information with the likelihood via Bayes theorem to obtain the posterior distribution. In the hybrid frequentist‐Bayesian approach followed in the present article, the analysis prior is not considered. Note also that, in general, the design prior must be proper to guarantee the existence of the expected values that we are about to introduce. For motivation and discussion on the distinction between these two priors, see Brutti et al. (2014) and references therein.

By taking the expected value of $U_{n}\left(\Theta \right)$ with respect to π(·), we obtain the Bayes utility of $\delta_{n}$

(5) $$u_{n}=E_{\pi }\left[U_{n}\left(\Theta \right)\right].$$

This quantity, called u‐PoS, is the one we propose to use as PoS. Comparisons with other definitions are discussed in Section 2.1.

To have a deeper insight into $u_{n}$, without loss of generality let us consider the one‐sided testing setting

(6) $$H_{0}:\theta \leq \theta_{0}\;\;\text{vs.}\;\;H_{1}:\theta >\theta_{0},$$

where $\theta_{0}$ is a suitable value for the unknown parameter; then $\Omega_{0}=\left(-\infty ,\theta_{0}\right]$ and $\Omega_{1}=\left(\theta_{0},\infty \right)$. Assume that Θ is a continuous random variable with design prior π(·). This setup is typically used for superiority trials that will be considered in the following application (Section 3), but extensions to other types of hypotheses are straightforward.

From (2), it follows that

$$\begin{array}{ccc} u_{n} & = & E_{\pi }\left[\left(1-\alpha_{n}\left(\Theta \right)\right)I_{\Omega_{0}}\left(\Theta \right)+\left(1-\beta_{n}\left(\Theta \right)\right)I_{\Omega_{1}}\left(\Theta \right)\right]=\\ & = & \int_{\Omega }\left(1-\alpha_{n}\left(\theta \right)\right)I_{\Omega_{0}}\left(\theta \right)\pi \left(\theta \right)d\theta +\int_{\Omega }\left(1-\beta_{n}\left(\theta \right)\right)I_{\Omega_{1}}\left(\theta \right)\pi \left(\theta \right)d\theta . \end{array}$$

Now, let

(7) $${\tilde{\pi }}_{i}\left(\theta \right)=\frac{1}{p_{i}}\pi \left(\theta \right)I_{\Omega_{i}}\left(\theta \right)$$

be the conditional distribution of Θ on $\Omega_{i}$, where $p_{i}=\int_{\Omega_{i}}\pi \left(\theta \right)d\theta$, i=0,1. Noting that $I_{\Omega_{i}}\left(\theta \right)\pi \left(\theta \right)={\tilde{\pi }}_{i}\left(\theta \right)p_{i}$, we have

(8) $$\begin{array}{ccc} u_{n} & = & p_{0}\int_{\Omega_{0}}\left[1-\alpha_{n}\left(\theta \right)\right]\;{\tilde{\pi }}_{0}\left(\theta \right)d\theta +p_{1}\int_{\Omega_{1}}\left[1-\beta_{n}\left(\theta \right)\right]\;{\tilde{\pi }}_{1}\left(\theta \right)d\theta =\\ & = & p_{0}\;E_{{\tilde{\pi }}_{0}}\left[1-\alpha_{n}\left(\Theta \right)\right]+p_{1}\;E_{{\tilde{\pi }}_{1}}\left[1-\beta_{n}\left(\Theta \right)\right]\\ & = & p_{0}\;u_{n}^{0}+p_{1}\;u_{n}^{1}, \end{array}$$

where

(9) $$u_{n}^{0}=E_{{\tilde{\pi }}_{0}}\left[1-\alpha_{n}\left(\Theta \right)\right]\;\text{and}\;u_{n}^{1}=E_{{\tilde{\pi }}_{1}}\left[1-\beta_{n}\left(\Theta \right)\right]$$

are Bayes utilities of $\delta_{n}$ under $H_{0}$ and $H_{1}$, respectively.

Before moving on, notice that so far we have considered utility, expected utility, and Bayes utility of the decision function $\delta_{n}$. However, in the decision‐theoretic framework, statistical problems are typically formalized in terms of loss functions, risk functions, and Bayes risk (Parmigiani and Inoue 2009). It is straightforward to rephrase our problem in these terms, as follows:

$$\begin{array}{ccc} \text{loss function of}\;a_{i}: & L(\theta ,a_{i}) & =1-U\left(\theta ,a_{i}\right)=I_{\Omega_{j}}\left(\theta \right),\;j\neq i=0,1;\\ \text{risk function of}\;\delta_{n}: & R(\theta ,\delta_{n}) & =E_{f}\left[L(\theta ,\delta \left(X_{n}\right))\right]=R_{n}\left(\theta \right)=1-U_{n}\left(\theta \right);\\ \text{Bayes risk of}\;\delta_{n}: & r_{n} & =E_{\pi }\left[R_{n}\left(\Theta \right)\right]=1-u_{n}. \end{array}$$

### 2.1 Probabilities of Success: Alternative Definitions and Relationships

In the literature, the definition of the PoS is not univocal. For instance, Kunzmann et al. (2021) collect and review several previous proposals. Among them, we select the following three definitions and we compare them with u‐PoS.

**a**. PoS is defined as the joint probability of rejecting $H_{0}$ and the true treatment effect belonging to $\Omega_{1}$, that is,
$$\begin{array}{ccc} e_{n}^{a} & = & P[X_{n}\in X_{1},\Theta \in \Omega_{1}]\\ & = & P\left[\Theta \in \Omega_{1}\right]\times P\left[X_{n}\in X_{1}|\Theta \in \Omega_{1}\right]\\ & = & p_{1}\int_{\Omega_{1}}P_{f}\left[X_{n}\in X_{1}|H_{1}\right]{\tilde{\pi }}_{1}\left(\theta \right)d\theta \\ & = & \int_{\Omega }\eta_{n}\left(\theta \right)I_{\Omega_{1}}\left(\theta \right)\pi \left(\theta \right)d\theta \\ & = & \int_{\Omega_{1}}\eta_{n}\left(\theta \right)\pi \left(\theta \right)d\theta \\ & = & \int_{\Omega_{1}}\left[1-\beta_{n}\left(\theta \right)\right]\pi \left(\theta \right)d\theta \\ & = & p_{1}u_{n}^{1}, \end{array}$$
  where $u_{n}^{1}$ is defined by (9). From the fourth equality, it follows that $e_{n}^{a}$ is the average probability to reject $H_{0}$ under $H_{1}$, weighted with the unconditional prior density. As far as we know, this quantity has been introduced by Spiegelhalter and Freedman (1986) and later on discussed and used, for instance, in Spiegelhalter et al. (2004), Shao et al. (2008), Liu (2010), Gasparini et al. (2013), Ciarleglio et al. (2015), and Kunzmann et al. (2021).
**b**. PoS is defined as the expected value of the probability to reject $H_{0}$ with respect to the prior density conditional on $H_{1}$, that is,
$$\begin{array}{ccc} e_{n}^{b} & = & E_{\pi }\left[\eta_{n}\left(\Theta \right)|\Theta \in \Omega_{1}\right]=\int_{\Omega_{1}}\eta_{n}\left(\theta \right){\tilde{\pi }}_{1}\left(\theta \right)d\theta \\ & = & \int_{\Omega_{1}}\left(1-\beta_{n}\left(\theta \right)\right){\tilde{\pi }}_{1}\left(\theta \right)d\theta =u_{n}^{1}. \end{array}$$
  Therefore, $e_{n}^{b}=e_{n}^{a}/p_{1}$. See Spiegelhalter and Freedman (1986), Spiegelhalter et al. (2004), and Liu (2010), and the discussion in Kunzmann et al. (2021) on the relationships between $e_{n}^{a}$ and $e_{n}^{b}$.
**c**. PoS is defined as the marginal probability to reject $H_{0}$, with respect to the design prior π(·) defined over the entire parameter space, that is
$$\begin{array}{ccc} e_{n}^{c} & = & E_{\pi }\left[\eta_{n}\left(\Theta \right)\right]=\int_{\Omega }\eta_{n}\left(\theta \right)\pi \left(\theta \right)d\theta \\ & = & p_{0}\int_{\Omega_{0}}\alpha_{n}\left(\theta \right)\;{\tilde{\pi }}_{0}\left(\theta \right)d\theta +p_{1}\int_{\Omega_{1}}\left(1-\beta_{n}\left(\theta \right)\right)\;{\tilde{\pi }}_{1}\left(\theta \right)d\theta \\ & = & p_{0}\;\left(1-u_{n}^{0}\right)+p_{1}\;u_{n}^{1}, \end{array}$$
  where $u_{n}^{0}$ and $u_{n}^{1}$ are defined by (9). This quantity is the assurance introduced by O'Hagan and Stevens (2001) and discussed in O'Hagan et al. (2005). The above decomposition, also discussed in Liu (2010) and Kunzmann et al. (2021), shows that $e_{n}^{c}$ mixes up expected values of the type I error function $\alpha_{n}\left(\theta \right)$ and of the expected utility $\left[1-\beta_{n}\left(\theta \right)\right]$. As noted, for instance, by Spiegelhalter et al. (2004) and Kunzmann et al. (2021), if $p_{0}$ is negligible, $e_{n}^{c}\approx e_{n}^{a}$ and, therefore, $e_{n}^{c}$ can be used as an approximation to $e_{n}^{a}$ in many practical situations.

All the quantities $e_{n}^{j}$, j=a,b,c, and $u_{n}$ can be expressed as expected values with respect to π(·):

$$\begin{array}{ccc} e_{n}^{a} & = & E_{\pi }\left[\eta_{n}\left(\Theta \right)I_{\Omega_{1}}\left(\Theta \right)\right],\\ e_{n}^{b} & = & E_{\pi }\left[\eta_{n}\left(\Theta \right)I_{\Omega_{1}}\left(\Theta \right)\right]/p_{1},\\ \;e_{n}^{c} & = & E_{\pi }\left[\eta_{n}\left(\Theta \right)\right],\\ u_{n} & = & E_{\pi }\left[\left(1-\eta_{n}\left(\Theta \right)\right)I_{\Omega_{0}}\left(\Theta \right)+\eta_{n}\left(\Theta \right)I_{\Omega_{1}}\left(\Theta \right)\right]. \end{array}$$

We can summarize the relationships between $u_{n}$ and the three other quantities in the following remarks:

1. for any design prior π(·) on Ω,
(10) $$\begin{array}{ccc} e_{n}^{a} & = & p_{1}u_{n}^{1},\\ e_{n}^{b} & = & u_{n}^{1},\\ e_{n}^{c} & = & p_{0}\;\left(1-u_{n}^{0}\right)+p_{1}\;u_{n}^{1}; \end{array}$$
2. if π(·) is a point‐mass prior on $\theta_{d}\in \Omega_{1}$, then $e_{n}^{a}=e_{n}^{b}=e_{n}^{c}=u_{n}$ coincide with $\eta_{n}\left(\theta_{d}\right)$;
3. if $p_{0}=0$, then $e_{n}^{a}=e_{n}^{b}=e_{n}^{c}=u_{n}^{1}=u_{n}$;
4. if $p_{0}>0$, then $e_{n}^{c}$ is the weighted average of Bayes risk under $H_{0}$ and Bayes utility under $H_{1}$, whereas $u_{n}$ (u‐PoS) is the weighted average of Bayes utilities under $H_{0}$ and $H_{1}$, respectively;
5. for $p_{0}\in \left(0,1\right)$, $u_{n}>e_{n}^{a}$; $u_{n}>e_{n}^{c}$ if and only if $u_{n}^{0}>\frac{1}{2}$; $e_{n}^{a}<e_{n}^{b}$; $e_{n}^{a}<e_{n}^{c}$.

The distribution of the random variables that appear in the definition of the above expected values are studied in Mariani et al. (2024).

### 2.2 Numerical Computation

Closed‐form expressions of $u_{n}$ and $e_{n}^{j}$, j=a,b,c are not in general available. To compute their numerical values, one can resort to Monte Carlo approximation which requires only the explicit expression of $\eta_{n}\left(\theta \right)$ and draws $\theta^{\left(r\right)}$, r=1,…,M from the design prior π(·). Specifically, we have that

$$\begin{array}{ccc} e_{n}^{a} & = & E_{\pi }\left[\eta_{n}\left(\Theta \right)I_{\Omega_{1}}\left(\Theta \right)\right]\approx \frac{1}{M}\sum_{r=1}^{M}\eta_{n}\left(\theta^{\left(r\right)}\right)I_{\Omega_{1}}\left(\theta^{\left(r\right)}\right),\\ e_{n}^{b} & = & E_{\pi }\left[\eta_{n}\left(\Theta \right)|\Theta \in \Omega_{1}\right]\approx \frac{1}{{\bar{p}}_{1}}\frac{1}{M}\sum_{r=1}^{M}\eta_{n}\left(\theta^{\left(r\right)}\right)I_{\Omega_{1}}\left(\theta^{\left(r\right)}\right),\;\;\\ {\bar{p}}_{1} & = & \frac{1}{M}\sum_{r=1}^{M}I_{\Omega_{1}}\left(\theta^{\left(r\right)}\right),\\ e_{n}^{c} & = & E_{\pi }\left[\eta_{n}\left(\Theta \right)\right]\approx \frac{1}{M}\sum_{r=1}^{M}\eta_{n}\left(\theta^{\left(r\right)}\right),\\ u_{n} & = & E_{\pi }\left[U_{n}\left(\Theta \right)\right]\\ & & \approx \frac{1}{M}\sum_{r=1}^{M}\left\{\left[1-\eta_{n}\left(\theta^{\left(r\right)}\right)\right]I_{\Omega_{0}}\left(\theta^{\left(r\right)}\right)+\eta_{n}\left(\theta^{\left(r\right)}\right)I_{\Omega_{1}}\left(\theta^{\left(r\right)}\right)\right\}. \end{array}$$

In Section 3.1, this algorithm is used for the normal model with several design priors. Code is implemented in R (R Core Team 2021). To guarantee reproducibility of results and guide practitioners toward a more informed use of $u_{n}$ and $e_{n}^{j}$, j=a,b,c, we also provide a shiny app (Chang et al. 2022), called uPoS available at https://6kp5ow‐francesco‐mariani.shinyapps.io/uPoS/.

Note that, even in the case of the normal model with known variance with conjugate design priors, the only explicit formula is for $e_{n}^{c}$ (see Equation (14)). An alternative way to obtain the expected values $u_{n}$ and $e_{n}^{j}$, j=a,b,c is direct numerical integration of the density functions of the corresponding random variables (Mariani et al. 2024).

### 2.3 Sample Size Determination

The Bayes utility $u_{n}$ can be used to define the following sample size criterion:

(11) $$n_{u}^{★}=\min\left\{n\in N:u_{n}\geq \varepsilon_{u}\right\},$$

where $\varepsilon_{u}\in \left(0,1\right)$. Similar criteria can be defined using each quantity $e_{n}^{j}$, that is,

(12) $$n_{j}^{★}=\min\left\{n\in N:e_{n}^{j}\geq \varepsilon_{j}\right\},\;j=a,b,c,$$

where $\varepsilon_{j}\in \left(0,1\right)$. In order to obtain feasible values of $n_{j}^{★}$, it is necessary to set appropriate thresholds $\varepsilon_{j}$, for j=a,b,c,u. For this reason, we need to find the limiting values $u_{\infty }=\lim_{n\to \infty }u_{n}$ and $e_{\infty }^{j}=\lim_{n\to \infty }e_{n}^{j}$, j=a,b,c. These values can be easily determined under the assumption of size‐α consistency for the test (van der Vaart 1998) that is expressed by the following definition.

Definition 2.1 (size‐α consistency) For any given α∈(0,1), a test is size‐α consistent if, for all θ∈Ω, the sequence $\left(\eta_{n}\left(\theta \right);n\in N\right)$ converges to the limiting power function defined as

(13) $$\eta_{\infty }\left(\theta \right)=\lim_{n\to \infty }\eta_{n}\left(\theta \right)=\left\{\begin{array}{cc} 0 & \;\theta \in \Omega_{0}\setminus \left\{\theta_{0}\right\},\\ \alpha & \;\theta =\theta_{0},\\ 1 & \;\theta \in \Omega_{1}. \end{array}\right.\;$$

For condition (13),

$$\left\{\begin{array}{cc} \alpha_{n}\left(\theta \right)\to 0 & \;\theta \in \Omega_{0}\setminus \left\{\theta_{0}\right\},\\ \beta_{n}\left(\theta \right)\to 0 & \;\theta \in \Omega_{1}, \end{array}\right.$$

which, recalling (2), implies that $U_{n}\left(\theta \right)\to 1$ for all $\theta \in \Omega \setminus \{\theta_{0}\}$. From the dominated convergence theorem,

$$\lim_{n\to \infty }u_{n}^{0}=\lim_{n\to \infty }E_{{\tilde{\pi }}_{0}}\left[1-\alpha_{n}\left(\Theta \right)\right]=E_{{\tilde{\pi }}_{0}}\left(\lim_{n\to \infty }\left[1-\alpha_{n}\left(\Theta \right)\right]\right)=1,$$

$$\lim_{n\to \infty }u_{n}^{1}=\lim_{n\to \infty }E_{{\tilde{\pi }}_{1}}\left[1-\beta_{n}\left(\Theta \right)\right]=E_{{\tilde{\pi }}_{1}}\left(\lim_{n\to \infty }\left[1-\beta_{n}\left(\Theta \right)\right]\right)=1,$$

and

$$\begin{array}{ccc} u_{\infty } & = & \lim_{n\to \infty }u_{n}=1. \end{array}$$

As a consequence of Equation (10),

$$e_{\infty }^{b}=u_{\infty }=1\;\text{and}\;e_{\infty }^{a}=e_{\infty }^{c}=p_{1}.$$

Therefore, if the design prior is such that $p_{0}>0$, then $n_{j}^{★}$ exists if and only if $\varepsilon_{j}<p_{1}$ for j=a,c; whereas $n_{b}^{★}$ and $n_{u}^{★}$ exist for any value of $\varepsilon_{b}$ and $\varepsilon_{u}$, regardless of the design prior.

## 3 Application to Clinical Trials: The Normal Case

For illustration, we consider the setup of the FREEDOM trial (Future Revascularization Evaluation in Patients with Diabetes Mellitus: Optimal Management of Multivessel Disease) published in Farkouh et al. (2012), where CABG is compared with PCI in diabetic patients with multivessel coronary artery disease. The original randomized trial design assigned patients to either CABG or PCI, who were followed for a minimum of 2 years. The primary outcome measure was a composite of death from any cause, nonfatal myocardial infarction, or nonfatal stroke: CABG was shown to be superior to PCI “in that it significantly reduced rates of death and myocardial infarction.” In a subsequent paper, Bittl and He (2017) commented on the results of the FREEDOM trial stating that “the finding of borderline lower mortality after CABG than after PCI at 5 years […] was not considered definitive […] and the trial was not powered for mortality” (p‐value = 0.049). Therefore, Bittl and He (2017) performed a Bayesian analysis where the prior distribution for the parameter of interest, the log odds ratio (log OR), was based on a meta‐analysis of eight historical trials and combined with the likelihood of the data of the FREEDOM trial.

We suppose now to plan a new phase III superiority trial based on a binary endpoint to compare the effects of CABG and PCI in terms of survival. As in Bittl and He (2017), we assume that trial data are summarized by the sample log OR with approximate normal distribution of parameters $\left(\theta ,\sigma^{2}/n\right)$, where θ is the log OR and $\sigma^{2}$ is assumed to be known. Following Spiegelhalter et al. (2004), we set $\sigma^{2}=4$ so that n can be interpreted as the effective number of observations in the trial, that is, the total number of events in the two arms. Referring to the hypotheses (6) with $\theta_{0}=0$, in the following sections we evaluate PoS of this new trial with u‐PoS and, for comparison, with the other quantities described in Section 2.1. Several alternative choices of the design priors for θ, based on historical data, are taken into account.

### 3.1 Unimodal Design Priors

As discussed before, the impact of the design prior on the different measures of PoS crucially depends on $p_{0}$, the probability assigned to $H_{0}$. Given $\theta_{d}$, the value used in the frequentist power analysis, we model uncertainty with three sets of design priors. For illustration, first of all we consider conventional normal priors, with support Ω that obviously yields $p_{0}>0$. In addition, we make use of a unimodal but not symmetric density, namely, a skew normal (see Azzalini 2013 for a comprehensive review). Finally, we adopt truncated normal densities where left truncation at $\theta_{0}=0$ implies that $p_{0}=0$.

- **Conventional normal prior:** the design prior for Θ is a normal density function of parameters $\left(\theta_{d},\sigma^{2}/n_{d}\right)$, where $n_{d}$ is the design prior sample size. Note that, in this case, at least the expression of $e_{n}^{c}$ can be given in closed form, that is,
(14) $$e_{n}^{c}=1-\Phi \left(\frac{\theta_{0}-\theta_{d}+\frac{\sigma }{\sqrt{n}}z_{1-\alpha }}{\xi_{d}}\right),$$
  where $\xi_{d}=\sigma \sqrt{1/n_{d}+1/n}$. For the other quantities, we resort to numerical computation as described in Section 2.2. In the following examples, we consider three levels of precision, by setting $n_{d}=15,46,165$, that yield different values of $p_{0}$. The case $n_{d}\to \infty$ corresponds to a point‐mass design prior and returns the frequentist power. By specifying three different values of $\theta_{d}$ based on historical data from Bittl and He (2017), we take into account the following scenarios:
  i. skeptical: $\theta_{d}=0.198$, the minimal clinical relevant effect difference;
  ii. intermediate: $\theta_{d}=0.372$, the midpoint between (i) and (iii);
  iii. enthusiastic: $\theta_{d}=0.545$, the guessed effect difference.
- **Skew normal prior**: the design prior for Θ is a skew normal density function of parameters $\left(\theta_{d},\sigma^{2}/n_{d},\lambda \right)$, where λ is the skewness parameter. We consider $\theta_{d}=0.198$ as in (i), the same values of $n_{d}$ as before and set λ=1. The choices of the parameters induce even smaller values of $p_{0}$.
- **Truncated normal prior:** the design prior for Θ is a truncated normal density function of parameters $\left(\theta_{d},\sigma^{2}/n_{d},\theta_{L},\theta_{U}\right)$, where $\left[\theta_{L},\theta_{U}\right]$ is the support of π(·). We consider $\theta_{d}=0.198$ as in (i), the same values of $n_{d}$ as before and set $\theta_{L}=\theta_{0}=0$ and $\theta_{U}=\infty$. The effect of left truncation on 0 is that $p_{0}=0$.

Table 1 reports the values of $u_{n}$ and of $e_{n}^{j}$, j=a,b,c, for the set of prior scenarios described above, given n=100,500. For comparison, we also include the case of point‐mass priors. The main comments are the following.

1. As expected, for all the design priors and sample sizes under consideration, values of $u_{n}$ are larger than those of $e_{n}^{a}$ and $e_{n}^{c}$. Even if $e_{n}^{a}<e_{n}^{c}$ by construction, their numerical values are always very similar, since $p_{0}\left(1-u_{n}^{0}\right)$ is almost negligible. Moreover, the values of $u_{n}$ and $e_{n}^{b}$ are very close.
2. Differences between $u_{n}$ and all the other quantities depend on $p_{0}$: the larger $p_{0}$, the more remarkable the differences, due to the contribution of $u_{n}^{0}$ in $u_{n}$.
3. For point‐mass design priors on $\theta_{d}\in \Omega_{1}$, $p_{0}=0$ and $u_{n}=e_{n}^{j}=\eta_{n}\left(\theta_{d}\right)$ for j=a,b,c. As expected, the value of $u_{n}$ increases with n and $\theta_{d}$.
4. For conventional normal priors, again, the higher $\theta_{d}$ and n, the larger the probabilities of success. As expected, for each combination of the design parameters, $u_{n}$ is the largest value. For larger values of $p_{0}$, induced by smaller values of $n_{d}$, differences between $u_{n}$ and the other quantities are more remarkable. As shown in Section 2.3, this has an impact on the choice of the sample size.
5. Skew normal priors assign smaller probabilities to $H_{0}$ than the normal prior with the same values of $\theta_{d}$ and $n_{d}$. As a consequence, the values of $e_{n}^{j}$, for j=a,b,c, and $u_{n}$ increase and tend to be closer for fixed n.
6. For the truncated normal priors, setting $\theta_{L}=\theta_{0}=0$ implies $p_{0}=0$, which results in all coincident values of the probabilities of success for each given combination of the design parameters and of the sample size.

**TABLE 1:** Values of $e_{n}^{j}$, j=a,b,c, and $u_{n}$ for several design scenarios, with $\sigma^{2}=4$.

Design prior	$\theta_{d}$	$n_{d}$	$p_{0}$	n	$e_{n}^{a}$	$e_{n}^{b}$	$e_{n}^{c}$	$u_{n}$
	0.198	∞	0	100	0.256	0.256	0.256	0.256
				500	0.715	0.715	0.715	0.715
Point‐mass	0.372	∞	0	100	0.585	0.585	0.585	0.585
				500	0.994	0.994	0.994	0.994
	0.545	∞	0	100	0.860	0.860	0.860	0.860
				500	1	1	1	1
		15	0.35	100	0.403	0.621	0.406	0.746
				500	0.538	0.828	0.539	0.889
Normal	0.198	46	0.25	100	0.356	0.476	0.360	0.605
				500	0.568	0.758	0.570	0.819
		165	0.10	100	0.298	0.331	0.300	0.396
				500	0.606	0.674	0.607	0.705
		15	0.24	100	0.533	0.698	0.536	0.763
				500	0.662	0.866	0.663	0.903
Normal	0.372	46	0.10	100	0.543	0.606	0.545	0.645
				500	0.764	0.852	0.765	0.864
		165	0.008	100	0.566	0.571	0.566	0.575
				500	0.899	0.907	0.900	0.907
		15	0.15	100	0.652	0.764	0.654	0.797
				500	0.776	0.908	0.776	0.922
Normal	0.545	46	0.03	100	0.724	0.749	0.725	0.758
				500	0.898	0.928	0.899	0.933
		165	0.0002	100	0.797	0.798	0.797	0.798
				500	0.987	0.987	0.987	0.987
		15	0.12	100	0.627	0.715	0.629	0.743
				500	0.784	0.894	0.785	0.906
Skew normal	0.198	46	0.06	100	0.539	0.575	0.540	0.600
				500	0.793	0.846	0.794	0.857
λ=1		165	0.01	100	0.425	0.430	0.426	0.437
				500	0.810	0.818	0.810	0.820
		15	0	100	0.623	0.623	0.623	0.623
				500	0.828	0.828	0.828	0.828
Truncated normal	0.198	46	0	100	0.471	0.471	0.471	0.471
				500	0.751	0.751	0.751	0.751
$\theta_{L}=0,\theta_{U}=\infty$		165	0	100	0.335	0.335	0.335	0.334
				500	0.672	0.672	0.672	0.672

**FIGURE 1:**
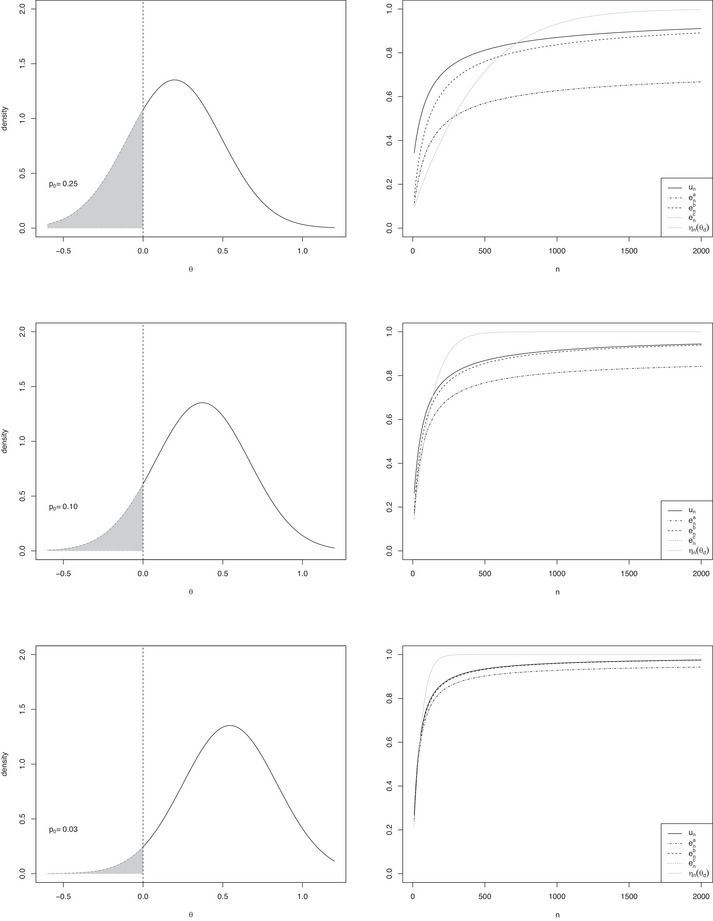
(*Left panels*) Plots of conventional normal prior densities centered on 0.198,0.372, and 0.545, respectively, with $n_{d}=46$. (*Right panels*) Plots of $\eta_{n}\left(\theta_{d}\right),e_{n}^{a},e_{n}^{b},e_{n}^{c}$, and $u_{n}$ as functions of n.

Figure 1 shows the plots of three conventional normal prior densities centered on 0.198, 0.372, and 0.545 respectively, with $n_{d}=46$ (left panels) and the corresponding plots of $\eta_{n}\left(\theta_{d}\right),e_{n}^{a},e_{n}^{b},e_{n}^{c},u_{n}$ as functions of n (right panels). The behavior of the curves is consistent with the previous remarks. In general, $\eta_{n}\left(\theta_{d}\right)$ converges to 1 more quickly than $u_{n}$ and $e_{n}^{b}$. For each n, the values of $u_{n}$ are uniformly, but slightly larger than $e_{n}^{b}$; their difference decreases as $\theta_{d}$ increases. Finally, $e_{n}^{a}$ and $e_{n}^{c}$ are almost coincident and they both tend to $p_{1}<1$.

Table 2 reports the optimal sample sizes obtained using criteria of Section 2.3 with thresholds equal to 80% of the maximum reachable values of each quantity, that is, $\varepsilon_{u}=\varepsilon_{b}=0.8$ and $\varepsilon_{a}=\varepsilon_{c}=0.8p_{1}$. The sample sizes obtained for the point‐mass design priors, denoted by $n^{★}$, coincide for each row with those obtained using the standard criterion based on the power function $\eta_{n}\left(\theta_{d}\right)$: the larger $\theta_{d}$, the smaller $n_{j}^{★}$, for j=a,b,c,u. For all other design priors, $n_{u}^{★}$ is always smaller than $n_{j}^{★}$, for j=a,b,c, with a more remarkable difference when $p_{0}$ is larger.

**TABLE 2:** Optimal sample sizes $n_{j}^{★}$, j=a,b,c,u, for several design scenarios, given the thresholds $\varepsilon_{u}=\varepsilon_{b}=0.8$ and $\varepsilon_{a}=\varepsilon_{c}=0.8p_{1}$.

Design prior	$\theta_{d}$	$n_{d}$	$p_{0}$	$n_{a}^{★}$	$n_{b}^{★}$	$n_{c}^{★}$	$n_{u}^{★}$
	0.198	∞	0	631	631	631	631
Point‐mass	0.372	∞	0	179	179	179	179
	0.545	∞	0	84	84	84	84
		15	0.35	353	353	344	161
Normal	0.198	46	0.25	739	739	725	437
		165	0.10	1086	1086	1075	924
		15	0.24	235	235	230	139
Normal	0.372	46	0.10	293	293	290	255
		165	0.009	255	255	255	253
		15	0.15	138	138	136	104
Normal	0.545	46	0.03	131	131	131	126
		165	0.0002	100	100	100	100
		15	0.12	180	180	178	146
Skew normal	0.198	46	0.06	340	340	337	301
λ=1		165	0.01	437	437	436	433
		15	0	367	367	367	367
Truncated normal	0.198	46	0	717	717	717	717
$\theta_{L}=0,\theta_{U}=\infty$		165	0	1040	1040	1040	1040

Table 3 compares numerical values of $u_{n^{★}}$ with those of the alternative versions of PoS for $n^{★}=179$ that yields $\eta_{n^{★}}\left(0.372\right)=0.8$ (as reported in Table 2 second row). Several values of $\theta_{d}$ and $n_{d}$ are considered. All the differences $u_{n^{★}}-e_{n^{★}}^{j}$ are consistently positive and decrease as $n_{d}$ increases (and $p_{0}$ decreases); moreover, these differences are larger for the normal prior than for the skew normal case, whereas they disappear for the truncated normal design prior ($p_{0}=0$). When $\theta_{d}=0.372$, values of $u_{n}$ are relatively robust with respect to $n_{d}$, whereas for $\theta_{d}=0.198$ (which implies $n^{★}=631$, see Table 2 first row) the effect of $n_{d}$ is more remarkable and the values of $u_{n}$ are more variable.

**TABLE 3:** Values of $u_{n^{★}}$ with respect to $e_{n^{★}}^{j}$, j=a,b,c, computed at $n^{★}=179$ (such that $\eta_{n^{★}}\left(0.372\right)=0.80$), for different design priors with $\theta_{d}=\left(0.198,0.372\right)$ and several choices of $n_{d}$.

Design prior	$\theta_{d}$	$n_{d}$	$p_{0}$	$u_{n^{★}}$	$\left(u_{n^{★}}-e_{n^{★}}^{a}\right)$	$\left(u_{n^{★}}-e_{n^{★}}^{b}\right)$	$\left(u_{n^{★}}-e_{n^{★}}^{c}\right)$
		10	0.38	0.848	0.375	0.089	0.374
		20	0.33	0.780	0.329	0.108	0.327
Normal	0.198	50	0.24	0.675	0.233	0.092	0.230
		100	0.16	0.586	0.159	0.077	0.156
		200	0.08	0.481	0.080	0.044	0.078
		10	0.14	0.847	0.145	0.029	0.144
		20	0.11	0.796	0.108	0.024	0.107
Skew normal	0.198	50	0.06	0.712	0.058	0.017	0.057
λ=1		100	0.03	0.640	0.023	0.007	0.022
		200	0.01	0.568	0.006	0.002	0.006
		10	0	0.753	0	0	0
		20	0	0.681	0	0	0
Truncated normal	0.198	50	0	0.576	0	0	0
$\theta_{L}=0,\theta_{U}=\infty$		100	0	0.506	0	0	0
		200	0	0.444	0	0	0
		10	0.278	0.852	0.284	0.065	0.282
		20	0.203	0.803	0.205	0.053	0.203
Normal	0.372	50	0.094	0.751	0.091	0.023	0.090
		100	0.031	0.721	0.033	0.011	0.032
		200	0.004	0.734	0.005	0.002	0.005
		10	0.077	0.889	0.075	0.007	0.075
		20	0.041	0.871	0.042	0.007	0.041
Skew normal	0.372	50	0.009	0.857	0.008	0.000	0.007
λ=1		100	0.001	0.861	0.001	0.000	0.001
		200	0	0.862	0.000	0.000	0.000
		10	0	0.794	0	0	0
		20	0	0.754	0	0	0
Truncated normal	0.372	50	0	0.720	0	0	0
$\theta_{L}=0,\theta_{U}=\infty$		100	0	0.709	0	0	0
		200	0	0.736	0	0	0

So far we have assumed that the variance $\sigma^{2}$ is known and equal to 4. However, this assumption can be relaxed. As an example, Table 4 reports the values of $e_{n}^{a},e_{n}^{b},e_{n}^{c},u_{n}$ for $\theta_{d}=0.198$ and $n_{d}=15,46,165$, that are obtained assuming an inverse gamma prior on $\sigma^{2}$ with expected value 4 and standard deviation equal to 1, namely, $\sigma^{2}∼\text{IG}\left(16,60\right)$. Interestingly, the probabilities $p_{0}$ and all the values in Table 4 are substantially equal to those of the second block of Table 1. Numerical exploration hints that these values are only affected by the expected value of $\sigma^{2}$ and are “insensitive to information about the variance,” as formally shown by Verdinelli (2000).

**TABLE 4:** Values of $e_{n}^{j}$, j=a,b,c, and $u_{n}$ for a normal design prior centered on $\theta_{d}=0.198$, assuming unknown variance, $\sigma^{2}∼\text{IG}\left(16,60\right)$.

Design prior	$\theta_{d}$	$n_{d}$	$p_{0}$	n	$e_{n}^{a}$	$e_{n}^{b}$	$e_{n}^{c}$	$u_{n}$
		15	0.35	100	0.403	0.622	0.406	0.752
				500	0.534	0.832	0.535	0.891
Normal	0.198	46	0.25	100	0.352	0.472	0.356	0.602
				500	0.566	0.753	0.568	0.812
		165	0.10	100	0.304	0.340	0.307	0.407
				500	0.621	0.690	0.623	0.719

### 3.2 Mixture of Normal Design Priors

In some experimental contexts, it might be useful to model prior uncertainty using multimodal densities. One case is when individual priors are pooled to obtain a *consensus prior* as in Crisp et al. (2018) and Dallow et al. (2018) that report several interesting case studies based on practical experience in pharmaceutical industry. Typically, in early phases of clinical development, the design prior simultaneously takes into account two scenarios: efficacy and nonefficacy of the treatment. Temple and Robertson (2021), for instance, consider a bimodal design prior, more specifically a mixture of two normal densities $\pi_{h}^{m}\left(\cdot \right)$ each centered on a value in $\Omega_{h}$, with weights $\omega_{h}$, h=0,1, that is, $\pi \left(\theta \right)=\omega_{0}\pi_{0}^{m}\left(\theta \right)+\left(1-\omega_{0}\right)\pi_{1}^{m}\left(\theta \right)$.

**FIGURE 2:**
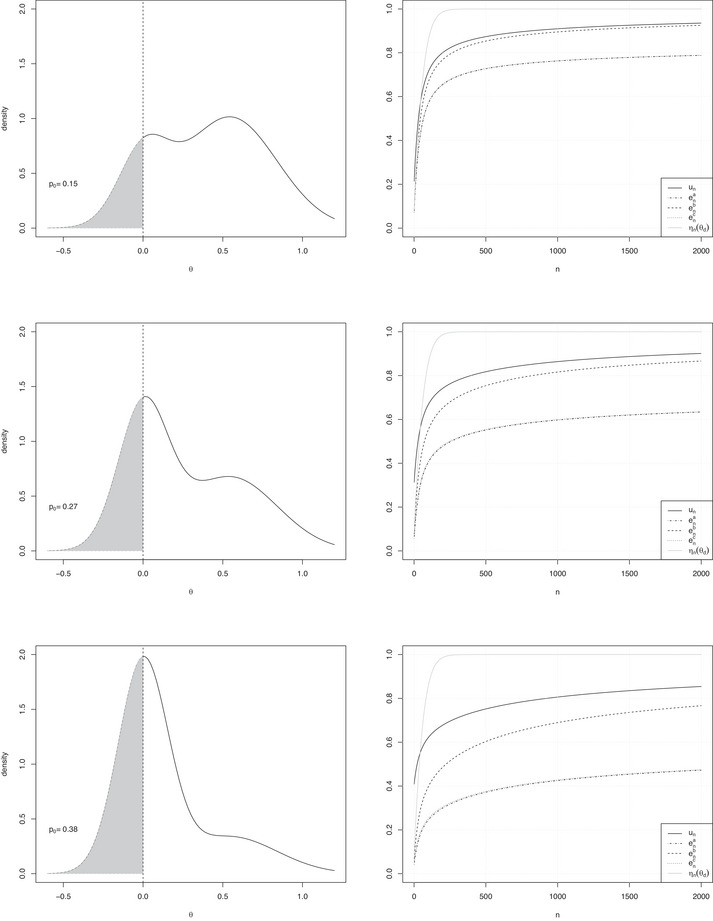
(*Left panels*) Plots of densities of mixtures of two normal priors ($\pi_{0}^{m}\left(\cdot \right)$ centered on $\theta_{0}=0$ with $n_{d}=165$ and $\pi_{1}^{m}\left(\cdot \right)$ centered on 0.545 with $n_{d}=46$) and weights, respectively, (i) $w_{0}=0.25,w_{1}=0.75$; (ii) $w_{0}=w_{1}=0.5$; and (iii) $w_{0}=0.75,w_{1}=0.25$. (*Right panels*) Plots of $\eta_{n}\left(\theta_{d}\right),e_{n}^{a},e_{n}^{b},e_{n}^{c},u_{n}$ as functions of n.

As an example, we consider three mixtures of two normal priors of parameters $\left(\theta_{d_{h}},\sigma^{2}/n_{d_{h}}\right)$, h=0,1: $\pi_{0}^{m}\left(\cdot \right)$ is centered on $\theta_{0}=0$ with $n_{d_{0}}=165$; $\pi_{1}^{m}\left(\cdot \right)$ is centered on 0.545 with $n_{d_{1}}=46$. We consider three sets of weights: (i) $w_{0}=0.25,w_{1}=0.75$; (ii) $w_{0}=w_{1}=0.5$; and (iii) $w_{0}=0.75,w_{1}=0.25$. Table 5 shows the values of $p_{0}$ and of $e_{n}^{a},e_{n}^{b},e_{n}^{c},u_{n}$ for n=100,500. Figure 2 (left panels) shows the plots of the three densities; correspondingly, right panels show the plots of $\eta_{n}\left(\theta_{d}\right),e_{n}^{a},e_{n}^{b},e_{n}^{c},u_{n}$ as functions of n. Finally, Table 6 reports the optimal sample sizes.

**TABLE 5:** Values of $e_{n}^{a},e_{n}^{b},e_{n}^{c},u_{n}$, given n=100,500, using as design prior a mixture of two normal densities: $\pi_{0}^{m}\left(\cdot \right)$ centered on $\theta_{0}=0$ with $n_{d}=165$ and $\pi_{1}^{m}\left(\cdot \right)$ centered on 0.545 with $n_{d}=46$, with several choices of the weights $w_{0},w_{1}$.

$w_{0}$	$p_{0}$	n	$e_{n}^{a}$	$e_{n}^{b}$	$e_{n}^{c}$	$u_{n}$
0.25	0.15	100	0.565	0.664	0.568	0.712
		500	0.727	0.851	0.729	0.871
0.5	0.27	100	0.410	0.557	0.415	0.669
		500	0.550	0.751	0.553	0.815
0.75	0.38	100	0.249	0.404	0.256	0.626
		500	0.378	0.611	0.381	0.756

**TABLE 6:** Optimal sample sizes $n_{j}^{★}$, j=a,b,c,u, using as design prior a mixture of two normal densities: $\pi_{0}^{m}\left(\cdot \right)$ centered on $\theta_{0}=0$ with $n_{d}=165$ and $\pi_{1}^{m}\left(\cdot \right)$ centered on 0.545 with $n_{d}=46$, for several choices of the weights $w_{0},w_{1}$ and thresholds $\varepsilon_{u}=\varepsilon_{b}=0.8$, $\varepsilon_{a}=\varepsilon_{c}=0.8p_{1}$.

$w_{0}$	$p_{0}$	$n_{a}^{★}$	$n_{b}^{★}$	$n_{c}^{★}$	$n_{u}^{★}$
0.25	0.15	255	255	250	191
0.5	0.27	821	821	793	398
0.75	0.38	2604	2604	2531	865

Remarks are similar to those of the unimodal prior case (Section 3.1). In general, $u_{n}$ is always larger than the other quantities; moreover, the larger $p_{0}$, the more remarkable the difference between $u_{n}$ and $e_{n}^{b}$, which is uniformly but moderately larger than $e_{n}^{a}$ and $e_{n}^{c}$. In addition, the plots of Figure 2 (right panels) clearly show that all the $e_{n}^{j}$ curves increase quite slowly. This translates in an actual difficulty in fulfilling sample size criteria: in the considered example of Table 6, a number of observations as large as 2000 is not enough to reach the thresholds $\varepsilon_{j}$, j=a,b,c; whereas the sample size determination criterion based on u‐PoS yields feasible values of $n_{u}^{★}$.

## 4 Conclusions

The article proposes a new definition of PoS as the expected value of the probability of choosing the correct hypothesis in a test, with respect to a design prior distribution of the parameter under investigation. The goals of this article have been accomplished as follows:

1. *Decision‐theoretic justification*. The proposed definition is the result of a formal decision‐theoretic look at the problem, since it coincides with the Bayes utility of a test under standard 0−1 utilities assigned to the two possible actions.
2. *Uncontroversial definition*. The main difference between our approach and several previous ones is that the latter are all derived as suitable expected values of the power function, that is, of the probability of rejecting the null hypothesis, rather than of the probability of choosing the true hypothesis.
3. *Asymptotic behavior*. As shown in Section 2.3, u‐PoS converges to one regardless of the design prior.

In the article, we compare our u‐PoS to the standard power function and to three other hybrid methods, among which the well‐known assurance (O'Hagan et al. 2005). Our analysis enlightens the crucial role of the design prior. When we employ a point‐mass prior on a value $\theta_{d}$ belonging to the alternative hypothesis, the four definitions coincide with the value $\eta_{n}\left(\theta_{d}\right)$ of the standard power function. When the design prior assigns zero probability to the null hypothesis, then $u_{n}$ and $e_{n}^{j}$, j=a,b,c coincide, but their values are different from $\eta_{n}\left(\theta_{d}\right)$. When $p_{0}>0$, a scenario that is sometimes taken into account in real trials (Temple and Robertson 2021), $e_{n}^{c}$ mixes up expected values of $\alpha_{n}\left(\Theta \right)$ and of $1-\beta_{n}\left(\Theta \right)$, whereas $e_{n}^{a}$ and $e_{n}^{b}$ do not take into account the null hypothesis (i.e., $u_{n}^{0}$) at all. In these cases, among the quantities that we consider, u‐PoS is the only one that correctly accounts for the null hypothesis, since it is a weighted average of $u_{n}^{0}$ and of $u_{n}^{1}$. In other words, u‐PoS amends the other expected values when it is necessary. Numerical examples show the effect of this correction: when $p_{0}$ is negligible, the values of $u_{n}$ and those of $e_{n}^{j}$, j=a,b,c, are very close, whereas when $p_{0}$ is not negligible, PoS measured by $u_{n}$ is typically larger than that indicated by the other methods. This fact has a clear impact on optimal sample sizes: values of $n_{u}^{★}$ are typically quite smaller than those of $n_{j}^{★}$, j=a,b,c. Such a phenomenon is dramatic when bimodal design priors are employed (see Section 3.2), that is, in all those experimental settings where, even though the goal of the trial is to reject the null (proving efficacy of a new treatment), the possibility that the null is the true hypothesis is seriously contemplated by the design distribution. For interesting real examples, see also Dallow et al. (2018).

In describing the crucial role of the design prior, it is worth mentioning, once again, the different asymptotics of $u_{n}$ and $e_{n}^{j}$, j=a,b,c when $p_{0}>0$. In these cases, $e_{n}^{a}$ and $e_{n}^{c}$ both tend to $p_{1}<1$: this feature has to be taken into account when fixing the thresholds $\varepsilon_{a}$ and $\varepsilon_{c}$ in sample size criteria. Conversely, $u_{n}$ and $e_{n}^{b}$ both tend to one regardless of the design prior. However, $u_{n}$ is typically larger than $e_{n}^{b}$, since the latter neglects the null hypothesis. As a consequence, values of $n_{u}^{★}$ can be substantially smaller than those of $n_{b}^{★}$ (see Table 2 and, for an extreme example, Table 6). Numerical examples of Sections 3.1 and 3.2 show that the impact of the prior is not only asymptotic, since the probability assigned to the null hypothesis as well as its shape are, in general, quite relevant.

In most of the examples considered in Section 3.1 the variance of the normal model is assumed to be known, in the modeling spirit of Spiegelhalter et al. (2004). Nevertheless, for one of the scenarios we have assigned a prior also to the variance, and we have checked the effects on $u_{n}$ and $e_{n}^{j}$, j=a,b,c. Numerical exploration suggests that it is only the mean of the prior assigned to the variance that matters, as discussed by Verdinelli (2000) for Bayesian designs in normal linear models.

In the present article, we focus on expected values of $U_{n}\left(\Theta \right)$ and of some other power‐related random variables. In a recent contribution, Mariani et al. (2024) study their overall probability distributions as proposed by Rufibach et al. (2016) for the power function. The analysis of these distributions can be used to detect the elements that affect PoS of a trial.

There are potential follow‐ups to the narration of the present article:

1. The proposed method is here implemented only for normal models, but different statistical models (for instance, for binary or count data) and other design priors could be utilized.
2. The focus of this work is on one‐sided hypotheses, but all the results of Section 2 can be adapted to all the typical testing setups used in clinical trials.
3. This article is entirely based on the use of the 0−1 utility function defined in (1). This choice implies a balanced attitude toward the two actions reflected by $U_{n}\left(\theta \right)$ in (2). Other utility functions could be considered in order to account for asymmetric consequences in accepting/rejecting the hypotheses, but in this way the direct interpretation of $U_{n}\left(\theta \right)$ and $u_{n}$ as probabilities could be lost.
4. In the pre‐experimental project of the trial, multiple parties are typically involved (e.g., trialist, data analyst, planner, …). Different utilities could be employed to incorporate several points of view in an adversarial risk analysis perspective. See, for instance, Etzioni and Kadane (1993), Brutti et al. (2014), and De Santis and Gubbiotti (2021).
5. In this work, we consider sample size determination criteria based on expected values of several alternative versions of PoS. Further criteria can be developed using other syntheses of the distribution of power‐related random variables (Mariani et al. 2024).
6. The approach proposed in the present article can be extended to a predictive setting by adjusting the definition of success of an experiment in terms of future data and then computing suitable predictive summaries of it. Similar considerations hold for interim power analysis. See, for instance, (Spiegelhalter et al. 2004, Section 6.6.3).

We hope to address these topics in future research.

## Supporting Information

Additional supporting information may be found in the online version of the article at the publisher's website.

## Conflicts of Interest

The authors declare no conflicts of interest.

## Open Research Badges

This article has earned an Open Data badge for making publicly available the digitally‐shareable data necessary to reproduce the reported results. The data is available in the Supporting Information section.

This article has earned an open data badge “**Reproducible Research**” for making publicly available the code necessary to reproduce the reported results. The results reported in this article could fully be reproduced.

## Supporting information


**Supporting File 1:** bimj70067‐sup‐0001‐Datacode.zip.

## Data Availability

Data sharing is not applicable to this article as no new data were created or analyzed in this study.
