# Supplementary material for: Rethinking Probability of Success as Bayes Utility
Source: Biom J. 2025 Jul 14;67(4):e70067. doi: 10.1002/bimj.70067 (PMC12260142; doi:10.1002/bimj.70067)
Supplement: Supplementary file 1 — Supporting File 1: bimj70067‐sup‐0001‐Datacode.zip. [file BIMJ-67-e70067-s001.zip › Code/reproduce_results.pdf]

Supplementary material :: Reproduction of results

Fulvio De Santis, Stefania Gubbiotti, Francesco Mariani

Rethinking probability of success as Bayes utility

Description

We replicate here the application study of **Section 3 - Application to clinical trials: the normal case** of the paper and report all the results provided in **Tables 1-6** and **Figures 1-2**.

```
source("uPoS_functions.R")
```

Tables

Table 1 Values of e_n^j, $j = a, b, c$ and u_n for several design scenarios, with $\sigma^2 = 4$, under different design prior assumptions:

- (rows 1:6) point-mass design prior, i.e. normal prior with $n_d = \infty$, for $\theta_d = 0.198, 0.372, 0, 545$
- (rows 7:24) normal design prior with $\theta_d = 0.198, 0.372, 0.545$ for $n_d = 15, 46, 165$ and $n = 100, 500$
- (rows 25:30) skew normal design prior with $\theta_d = 0.198$ for $n_d = 15, 46, 165$ and $\lambda = 1$
- (rows 31:36) truncated normal design prior with $\theta_d = 0.198$ for $n_d = 15, 46, 165$ and $\theta_L = 0, \theta_U = \infty$

	Design prior	th_d	n_d	pi0	n	eA	eB	eC	u
1	Point mass	0.198	Inf	0.00	100	0.256	0.256	0.256	0.256
2	Point mass	0.198	Inf	0.00	500	0.715	0.715	0.715	0.715
3	Point mass	0.372	Inf	0.00	100	0.585	0.585	0.585	0.585
4	Point mass	0.372	Inf	0.00	500	0.994	0.994	0.994	0.994
5	Point mass	0.545	Inf	0.00	100	0.860	0.860	0.860	0.860
6	Point mass	0.545	Inf	0.00	500	1.000	1.000	1.000	1.000
7	Normal	0.198	15	0.35	100	0.403	0.621	0.406	0.746
8	Normal	0.198	15	0.35	500	0.538	0.828	0.539	0.889
9	Normal	0.198	46	0.25	100	0.356	0.476	0.360	0.605
10	Normal	0.198	46	0.25	500	0.568	0.758	0.570	0.819
11	Normal	0.198	165	0.10	100	0.298	0.331	0.300	0.396
12	Normal	0.198	165	0.10	500	0.606	0.674	0.607	0.705
13	Normal	0.372	15	0.24	100	0.533	0.698	0.536	0.763
14	Normal	0.372	15	0.24	500	0.662	0.866	0.663	0.903
15	Normal	0.372	46	0.10	100	0.543	0.606	0.545	0.645
16	Normal	0.372	46	0.10	500	0.764	0.852	0.765	0.864
17	Normal	0.372	165	0.01	100	0.566	0.571	0.566	0.575
18	Normal	0.372	165	0.01	500	0.899	0.907	0.900	0.907
19	Normal	0.545	15	0.15	100	0.652	0.764	0.654	0.797
20	Normal	0.545	15	0.15	500	0.776	0.908	0.776	0.922
21	Normal	0.545	46	0.03	100	0.724	0.749	0.725	0.758
22	Normal	0.545	46	0.03	500	0.898	0.928	0.899	0.933
23	Normal	0.545	165	0.00	100	0.797	0.798	0.797	0.798
24	Normal	0.545	165	0.00	500	0.987	0.987	0.987	0.987
25	Skew Normal	0.198	15	0.12	100	0.627	0.715	0.629	0.743
26	Skew Normal	0.198	15	0.12	500	0.784	0.894	0.785	0.906
27	Skew Normal	0.198	46	0.06	100	0.539	0.575	0.540	0.600

	Design prior	th_d	n_d	pi0	n	eA	eB	eC	u
28	Skew Normal	0.198	46	0.06	500	0.793	0.846	0.794	0.857
29	Skew Normal	0.198	165	0.01	100	0.425	0.430	0.426	0.437
30	Skew Normal	0.198	165	0.01	500	0.810	0.818	0.810	0.820
31	Truncated Normal	0.198	15	0.00	100	0.623	0.623	0.623	0.623
32	Truncated Normal	0.198	15	0.00	500	0.828	0.828	0.828	0.828
33	Truncated Normal	0.198	46	0.00	100	0.471	0.471	0.471	0.471
34	Truncated Normal	0.198	46	0.00	500	0.751	0.751	0.751	0.751
35	Truncated Normal	0.198	165	0.00	100	0.335	0.335	0.335	0.335
36	Truncated Normal	0.198	165	0.00	500	0.672	0.672	0.672	0.672

Table 2 Optimal sample sizes $n_j^\star$, $j = a, b, c, u$, for several design scenarios, given the thresholds $\epsilon_u = \epsilon_b = 0.8$ and $\epsilon_a = \epsilon_c = 0.8p_1$.

- (rows 1:3) point-mass design prior, i.e. normal prior with $n_d = \infty$, for $\theta_d = 0.198, 0.372, 0, 545$
- (rows 4:12) normal design prior with $\theta_d = 0.198, 0.372, 0.545$ for $n_d = 15, 46, 165$
- (rows 13:15) skew normal design prior with $\theta_d = 0.198$ for $n_d = 15, 46, 165$ and $\lambda = 1$
- (rows 16:18) truncated normal design prior with $\theta_d = 0.198$ for $n_d = 15, 46, 165$ and $\theta_L = 0, \theta_U = \infty$

	Design prior	th_d	n_d	pi0	nA	nB	nC	nU
1	Point Mass	0.198	Inf	0.00	631	631	631	631
2	Point Mass	0.372	Inf	0.00	179	179	179	179
3	Point Mass	0.545	Inf	0.00	84	84	84	84
4	Normal	0.198	15	0.35	353	353	344	161
5	Normal	0.198	46	0.25	739	739	725	437
6	Normal	0.198	165	0.10	1086	1086	1075	924
7	Normal	0.372	15	0.24	235	235	230	139
8	Normal	0.372	46	0.10	293	293	290	255
9	Normal	0.372	165	0.01	255	255	255	253
10	Normal	0.545	15	0.15	138	138	136	104
11	Normal	0.545	46	0.03	131	131	131	126
12	Normal	0.545	165	0.00	100	100	100	100
13	Skew Normal	0.198	15	0.12	180	180	178	146
14	Skew Normal	0.198	46	0.06	340	340	337	301
15	Skew Normal	0.198	165	0.01	437	437	436	433
16	Truncated Normal	0.198	15	0.00	367	367	367	367
17	Truncated Normal	0.198	46	0.00	717	717	717	717
18	Truncated Normal	0.198	165	0.00	1040	1040	1040	1040

Table 3 Values of $u_{n^\star}$ wrt $e_{n^\star}$, $j = a, b, c$, computed at $n^\star = 179$ (such that $\eta_{n^\star}(0.372) = 0.80$, for different design priors with $\theta_d = 0.198, 0.372$ and several choices of n_d.

- (rows 1:5) normal design prior with $\theta_d = 0.198$ and (rows 16:20) $\theta_d = 0.545$ for $n_d = 10, 20, 50, 100, 200$ for $n^\star = 180$
- (rows 6:10) skew normal design prior with $\theta_d = 0.198$ and (rows 21:25) $\theta_d = 0.545$ for $n_d = 10, 20, 50, 100, 200$ and $\lambda = 1$
- (rows 11:15) truncated normal design prior with $\theta_d = 0.198$ and (rows 26:30) $\theta_d = 0.545$ for $n_d = 10, 20, 50, 100, 200$ and $\theta_L = 0, \theta_U = \infty$

	Design prior	th_d	n_d	pi0	u	u - eA	u - eB	u - eC
1	Normal	0.198	10	0.38	0.848	0.375	0.089	0.374
2	Normal	0.198	20	0.33	0.780	0.329	0.108	0.327
3	Normal	0.198	50	0.24	0.675	0.233	0.092	0.230
4	Normal	0.198	100	0.16	0.586	0.159	0.077	0.156
5	Normal	0.198	200	0.08	0.481	0.080	0.044	0.078
6	Skew normal	0.198	10	0.14	0.847	0.145	0.029	0.144
7	Skew normal	0.198	20	0.11	0.796	0.108	0.024	0.107
8	Skew normal	0.198	50	0.06	0.712	0.058	0.017	0.057
9	Skew normal	0.198	100	0.03	0.640	0.023	0.007	0.022
10	Skew normal	0.198	200	0.01	0.568	0.006	0.002	0.006
11	Truncated normal	0.198	10	0.00	0.753	0.000	0.000	0.000
12	Truncated normal	0.198	20	0.00	0.681	0.000	0.000	0.000
13	Truncated normal	0.198	50	0.00	0.576	0.000	0.000	0.000
14	Truncated normal	0.198	100	0.00	0.506	0.000	0.000	0.000
15	Truncated normal	0.198	200	0.00	0.444	0.000	0.000	0.000
16	Normal	0.372	10	0.28	0.852	0.284	0.065	0.282
17	Normal	0.372	20	0.20	0.803	0.205	0.053	0.203
18	Normal	0.372	50	0.09	0.751	0.091	0.023	0.090
19	Normal	0.372	100	0.03	0.721	0.033	0.011	0.032
20	Normal	0.372	200	0.00	0.734	0.005	0.002	0.005
21	Skew normal	0.372	10	0.08	0.889	0.075	0.007	0.075
22	Skew normal	0.372	20	0.04	0.871	0.042	0.007	0.041
23	Skew normal	0.372	50	0.01	0.857	0.008	0.000	0.007
24	Skew normal	0.372	100	0.00	0.861	0.001	0.000	0.001
25	Skew normal	0.372	200	0.00	0.862	0.000	0.000	0.000
26	Truncated normal	0.372	10	0.00	0.794	0.000	0.000	0.000
27	Truncated normal	0.372	20	0.00	0.754	0.000	0.000	0.000
28	Truncated normal	0.372	50	0.00	0.720	0.000	0.000	0.000
29	Truncated normal	0.372	100	0.00	0.709	0.000	0.000	0.000
30	Truncated normal	0.372	200	0.00	0.736	0.000	0.000	0.000

Table 4 Values of e_n^j, $j = a, b, c$ and u_n for a normal design prior centered on $\theta_d = 0.198$, assuming unknown variance, $\sigma^2 \sim IG(16, 60)$.

	Design prior	th_d	n_d	pi0	n	eA	eB	eC	u
1	Normal	0.198	15	0.35	100	0.403	0.622	0.406	0.752
2	Normal	0.198	15	0.36	500	0.534	0.832	0.535	0.891
3	Normal	0.198	46	0.25	100	0.352	0.472	0.356	0.602
4	Normal	0.198	46	0.25	500	0.566	0.753	0.568	0.812
5	Normal	0.198	165	0.11	100	0.304	0.340	0.307	0.407
6	Normal	0.198	165	0.10	500	0.621	0.690	0.623	0.719

Table 5 Values of e_n^j, $j = a, b, c$ and u_n given $n = 100, 500$, using as design prior a mixture of two normal densities: $\pi_0^m(\cdot)$ centered on $\theta_0 = 0$ with $n_d = 165$ and $\pi_1^m(\cdot)$ centered on 0.545 with $n_d = 46$, with several choices of the weights w_0, w_1.

	w_0	pi0	n	eA	eB	eC	u
1	0.25	0.15	100	0.565	0.664	0.568	0.712
2	0.25	0.15	500	0.727	0.851	0.729	0.871
3	0.50	0.26	100	0.410	0.557	0.415	0.669
4	0.50	0.27	500	0.550	0.751	0.553	0.815
5	0.75	0.38	100	0.249	0.404	0.256	0.626
6	0.75	0.38	500	0.378	0.611	0.381	0.756

Table 6 Optimal sample sizes $n_j^\star$, $j = a, b, c, u$, using as design prior a mixture of two normal densities: $\pi_0^m(\cdot)$ centered on $\theta_0 = 0$ with $n_d = 165$ and $\pi_1^m(\cdot)$ centered on 0.545 with $n_d = 46$, with several choices of the weights w_0, w_1 and with thresholds $\epsilon_u = \epsilon_b = 0.8$, $\epsilon_a = \epsilon_c = 0.8p_1$.

	w_0	pi0	nA	nB	nC	nU
1	0.25	0.15	255	255	250	191
2	0.50	0.27	821	821	793	398
3	0.75	0.38	2604	2604	2531	865

Figures

Figure 1 (Left panels) Plots of conventional normal prior densities centered on 0.198, 0.372, 0.545 respectively, with $n_d = 46$. (Right panels) Plots of $\eta_n(\theta_d)$, e_n^a, e_n^b, e_n^c, u_n as functions of n.

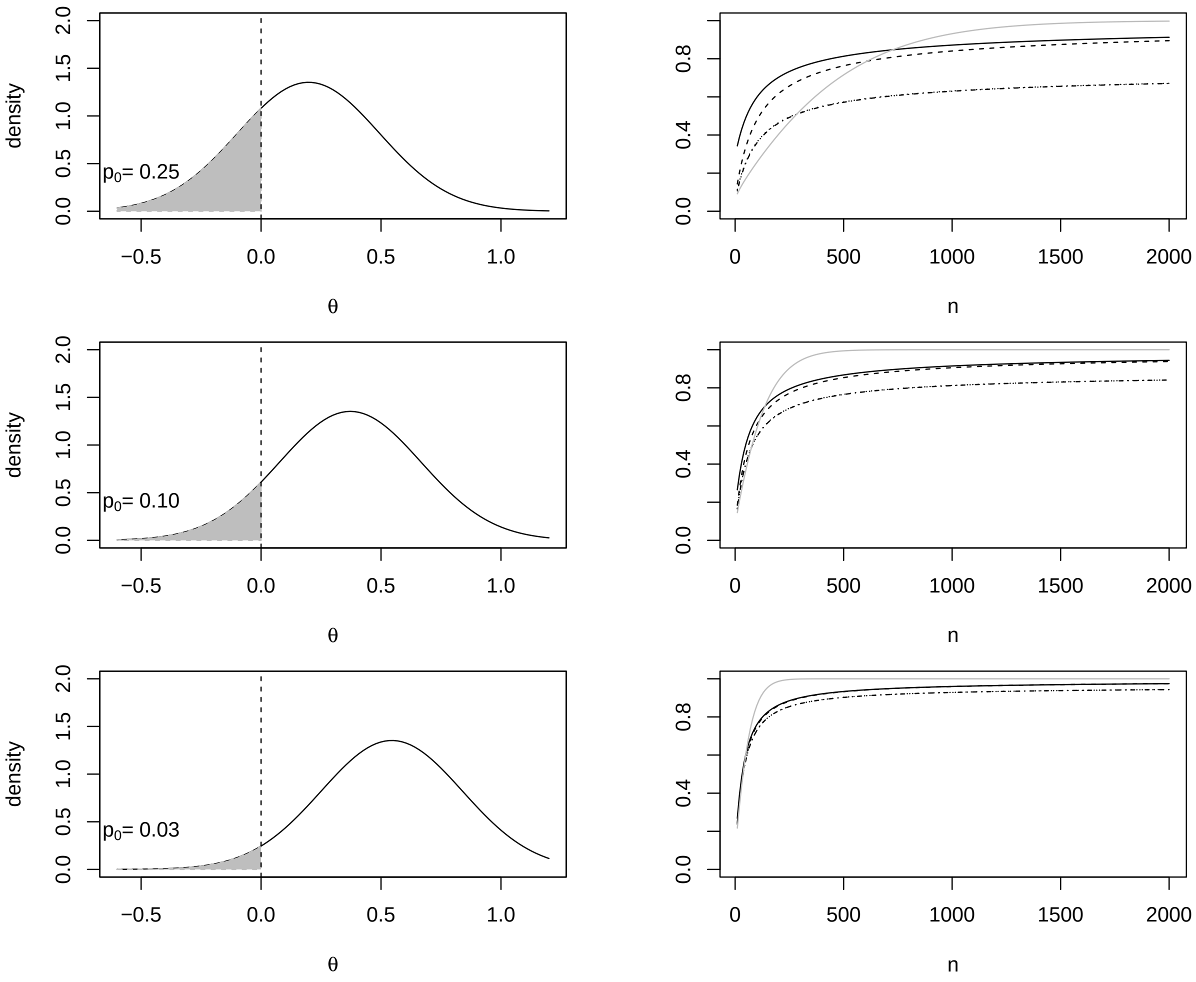

Figure 2 (Left panels) Plots of densities of mixtures of two normal priors $\pi_0^m(\cdot)$ centered on $\theta_0 = 0$ with $n_d = 165$ and $\pi_1^m(\cdot)$ centered on $\theta_0 = 0.545$ with $n_d = 46$ with weights respectively (i) $w_0 = 0.25$, $w_1 = 0.75$; (ii) $w_0 = w_1 = 0.5$; (iii) $w_0 = 0.75, w_1 = 0.25$. (Right panels) Plots of $\eta_n(\theta_d)$, e_n^a, e_n^b, e_n^c, u_n as functions of n.

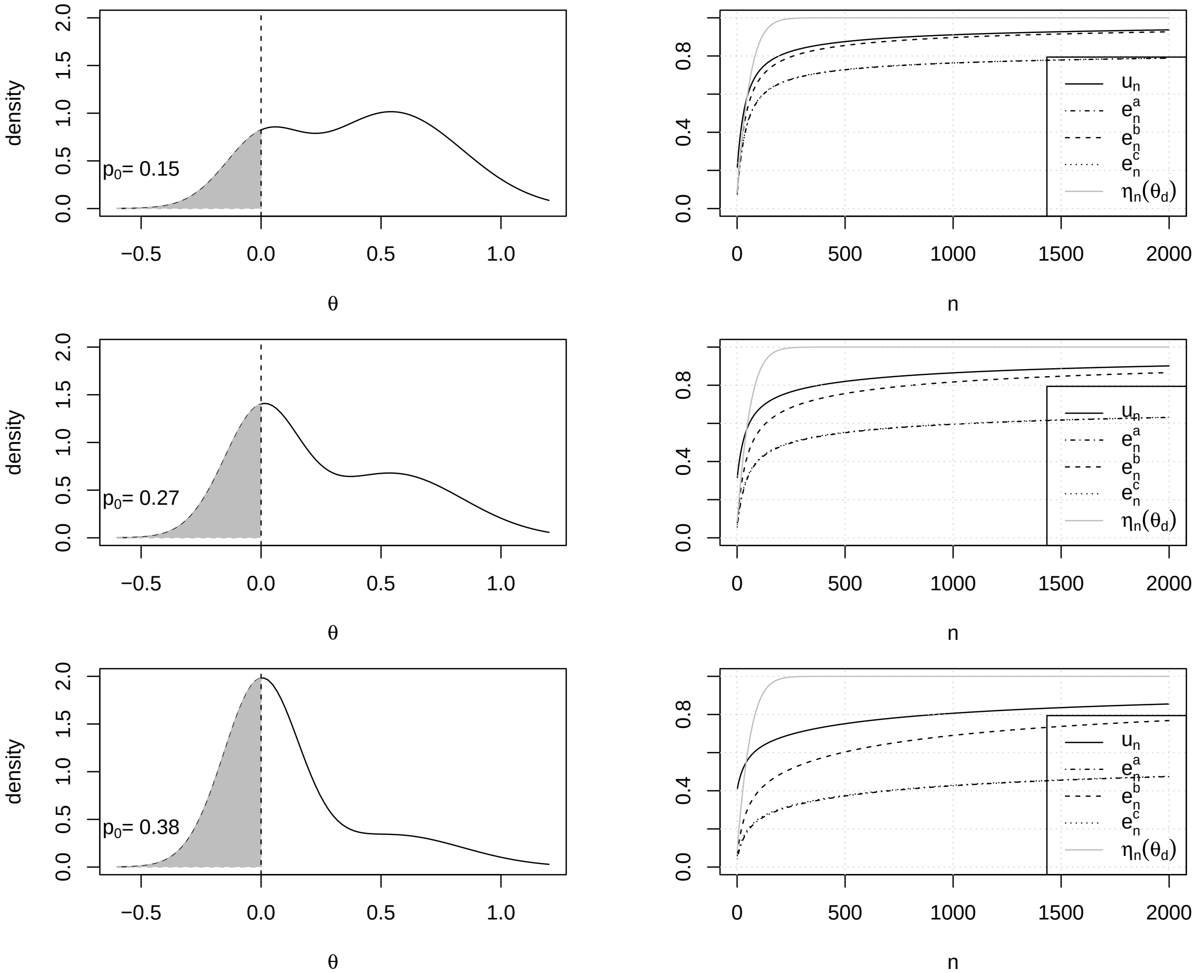

Session info:

```
R version 4.3.1 (2023-06-16)
Platform: aarch64-apple-darwin20 (64-bit)
Running under: macOS Ventura 13.1

Matrix products: default
BLAS:   /Library/Frameworks/R.framework/Versions/4.3-arm64/Resources/lib/libRblas.0.dylib
LAPACK: /Library/Frameworks/R.framework/Versions/4.3-arm64/Resources/lib/libRlapack.dylib;  [illegible]

locale:
[1] en_US.UTF-8/en_US.UTF-8/en_US.UTF-8/C/en_US.UTF-8/en_US.UTF-8

time zone: Europe/Rome
tzcode source: internal

attached base packages:
[1] stats4    stats     graphics  grDevices utils     datasets  methods
[8] base

other attached packages:
[1] knitr_1.44      invgamma_1.1    EnvStats_3.0.0  truncnorm_1.0-9
[5] sn_2.1.1

loaded via a namespace (and not attached):
 [1] digest_0.6.33       numDeriv_2016.8-1.1 fastmap_1.1.1
 [4] xfun_0.52           htmltools_0.5.8.1   rmarkdown_2.25
 [7] cli_3.6.4           mnormt_2.1.1        compiler_4.3.1
[10] rstudioapi_0.15.0   tools_4.3.1         evaluate_0.21
[13] yaml_2.3.7          rlang_1.1.6         jsonlite_1.8.7
```

```r
save.image(file = "uPoS.RData")
```
